# Intermittent Surface Oxygenation Results in Similar Mitochondrial Protection and Maintenance of Aerobic Metabolism as Compared to Continuous Oxygenation during Hypothermic Machine Kidney Machine Perfusion

**DOI:** 10.3390/jcm12113731

**Published:** 2023-05-29

**Authors:** Tom Darius, Martial Vergauwen, Louis Maistriaux, Robin Evrard, Andrea Schlegel, Matteo Mueller, Donna O’Neil, Andrew Southam, Selda Aydin, Arnaud Devresse, Martine De Meyer, Pierre Gianello, Christian Ludwig, Philipp Dutkowski, Michel Mourad

**Affiliations:** 1 Surgery and Abdominal Transplant Unit, University Clinics Saint Luc, Université Catholique de Louvain, 1200 Brussels, Belgium; arnaud.devresse@saintluc.uclouvain.be (A.D.); martine.demeyer@saintluc.uclouvain.be (M.D.M.); michel.mourad@saintluc.uclouvain.be (M.M.); 2 Institut de Recherche Expérimentale et Clinique (IREC), Pôle de Chirurgie Expérimentale et Transplantation, Université Catholique de Louvain, 1200 Brussels, Belgium; martial.vergauwen@uclouvain.be (M.V.); louis.maistriaux@uclouvain.be (L.M.); robin.evrard@uclouvain.be (R.E.); pierre.gianello@uclouvain.be (P.G.); 3 Institut de Recherche Expérimentale et Clinique (IREC), Pôle de Morphologie, Université Catholique de Louvain, 1200 Brussels, Belgium; 4 Institut de Recherche Expérimentale et Clinique (IREC), Neuro Musculo-Skeletal Laboratory (NMSK), Université Catholique de Louvain, 1200 Brussels, Belgium; 5 Department of Surgery and Transplantation, Swiss HPB Center, University Hospital Zurich, 8091 Zürich, Switzerland; schlegel.andrea@outlook.de (A.S.); matteo.mueller@usz.ch (M.M.); philipp.dutkowski@usz.ch (P.D.); 6 Phenome Centre Birmingham, College of Life and Environmental Sciences, University of Birmingham, Birmingham B15 2TT, UK; d.m.oneil@bham.ac.uk (D.O.); a.d.southam@bham.ac.uk (A.S.); 7 Department of Pathology, University Clinics Saint-Luc, Université Catholique de Louvain, 1200 Brussels, Belgium; selda.aydin@saintluc.uclouvain.be; 8 Department of Nephrology, University Clinics Saint-Luc, Université Catholique de Louvain, 1200 Brussels, Belgium; 9 Institute of Metabolism and Systems Research (IMSR), College of Medical and Dental Sciences, University of Birmingham, Birmingham B15 2TT, UK; c.ludwig@bham.ac.uk

**Keywords:** hypothermic machine perfusion, kidney preservation, oxygenation, mitochondria, organ preservation, kidney assessment

## Abstract

Short bubble and subsequent surface oxygenation is an innovative oxygenation technique and alternative for membrane oxygenation during hypothermic machine perfusion (HMP). The metabolic effect of the interruption of surface oxygenation for 4 h (mimicking organ transport) during HMP was compared to continuous surface and membrane oxygenation in a pig kidney ex situ preservation model. After 30 min of warm ischemia by vascular clamping, a kidney of a ±40 kg pig was procured and subsequently preserved according to one of the following groups: (1) 22-h HMP + intermittent surface oxygenation (*n* = 12); (2) 22-h HMP + continuous membrane oxygenation (*n* = 6); and (3) 22-h HMP + continuous surface oxygenation (*n* = 7). Brief perfusate O_2_ uploading before kidney perfusion was either obtained by direct bubble (groups 1, 3) or by membrane (group 2) oxygenation. Bubble oxygenation during minimum 15 min was as efficient as membrane oxygenation in achieving supraphysiological perfusate pO_2_ levels before kidney perfusion. Metabolic tissue analysis (i.e., lactate, succinate, ATP, NADH, and FMN) during and at the end of the preservation period demonstrated similar mitochondrial protection between all study groups. Short bubble and subsequent intermittent surface oxygenation of the perfusate of an HMP-kidney might be an effective and cheap preservation strategy to protect mitochondria, eliminating the need/costs of a membrane oxygenator and oxygen source during transport.

## 1. Introduction

Preclinical studies demonstrated the protective effect of active oxygen supply during hypothermic machine perfusion (HMP) of kidneys [1,2,3,4,5,6]. Its effect is superior when machine perfusion is started from the moment of organ procurement until transplantation as compared to different end-ischemic preservation strategies [6]. In addition, high oxygen concentrations during HMP resulted in a superior support of aerobic metabolism [1,7,8,9]. This was recently confirmed in a multicenter randomized clinical trial (RCT) from kidneys originated from donation after circulatory death (DCD), Maastricht category III, over 50 years. Patients were randomized to receive a kidney following either oxygenated (HMPO_2_) or non-oxygenated HMP. This double-blinded study contained 106 kidney pairs perfused during the entire preservation period. The primary outcome of 12-month estimated glomerular filtration rate (eGFR) was similar between both study groups. However, graft failure rate at one year after transplantation was significantly lower in the oxygenated group as compared to the non-oxygenated HMP group (3% versus 10%, *p* = 0.028) [10]. Two matched-case studies and one RCT were not able to confirm these positive effects of oxygen when applied as an end-ischemic preservation strategy for kidneys originated from extended criteria donors (ECD) after brain death (DBD). Under such conditions, there was no demonstrable improvement for delayed graft function (DGF), functional DGF, and primary nonfunction rate, nor early graft function and biopsy-proven acute rejection rate as compared to static cold storage (SCS) alone [11,12,13]. 

The duration of active oxygenation (continuous versus intermittent) during kidney HMP to achieve ‘ideal’ aerobic support and mitochondrial protection remains less explored. Our group demonstrated in a pig kidney auto-transplant model that O_2_ uploading for a short period of only 2 h at the start of HMP by membrane oxygenation results in a similar mitochondria protection as compared with continuous membrane oxygenation during the entire HMP period and a superior initial graft function as compared with briefly administrating oxygen only at the end of HMP preservation [14]. This illustrates that in DCD, kidneys correction of oxygen debt related to the procurement procedure needs to be corrected preferably at the start of HMP, and not at the end. 

Moreover, different oxygen administration techniques (classical membrane oxygenation versus alternative oxygenation techniques) during HMP remain underexplored. A feasibility study in a pig kidney DCD autotransplant model demonstrated that brief bubble and intermittent surface oxygenation during HMP (30 min at start and 1 h at the end of 22-h HMP) has the potential to be an alternative oxygenation technique to improve kidney function. This oxygenation strategy resulted in a similar functional outcome when compared with membrane-oxygenated HMP-kidneys [15]. In contrast to functional outcome, metabolic tissue analysis at the end of HMP was in favor of the membrane-oxygenated HMPO_2_ group [15]. This suggests that the optimal duration of surface oxygenation during HMP merits further exploration.

Therefore, this study evaluates the metabolic effect of a short interruption (4 h, mimicking organ transport) of surface oxygenation during 22 h of HMP as compared to continuous surface and membrane oxygenation in a pig kidney ex situ preservation model. In addition, real time concentration of pO2 in the perfusion fluid, renal resistance, and flow were measured, and biomarkers and metabolic profiles were analyzed from perfusate and tissue samples.

## 2. Materials and Methods

### 2.1. Animals

The experiments with the use of female Belgian Landrace pigs of ±40 kg were approved by the local Ethical Committee for Animal Care in accordance with the Belgian and European legislation (Directive-10-63/EU) and realized according to international guidelines and Belgian laws on experimental animal welfare.

### 2.2. Study Design

An ex situ preservation porcine kidney model was used based on previous experiences in the autotransplant setting [6,14,15].

### 2.3. Anesthetic and Surgical Protocol 

General anesthesia was realized by Zoletil^®^ and Rompun^®^ after induction by 6 mL/kg of Zoletil 100^®^ (Virbac, Carros, France) and 2 mg/kg of Rompun^®^ (Bayer, Leverkusen, Germany). Intravenous access was established through an ear vein. A Portex endotracheal tube (Hythe, Kent, UK) was inserted. After tracheal intubation, gas anesthesia was maintained by oxygen and Isoflurane (Forene^®^, AbbVie, Wavre, Belgium) (0–1.5%) under surveillance of cardiorespiratory monitoring (Datex-Ohmeda S/5).

After a preceding 30 min of warm ischemia induced by vascular clamping, both kidneys were procured and subsequently flushed with 200 mL of Kidney Perfusion Solution-1 (Organ Recovery Systems, Diegem, Belgium). The kidneys were preserved according to the three study groups: (1) 22-h HMP with intermittent surface oxygenation (30 min surface oxygenation at start followed by 4 h of interruption of active oxygenation and restarting active surface oxygenation from 4 h 30 until the end of HMP); (2) 22-h HMP with continuous membrane oxygenation; and (3) 22-h HMP with continuous surface oxygenation.

After the bilateral nephrectomy, a lethal intravenous injection of T61^®^ was administrated under general anesthesia (Intervet International; MSD Animal Health, Boxmeer, The Netherlands). 

### 2.4. Machine Perfusion 

Renal perfusion was realized by the LifePort Kidney Transporter^®^ (Organ Recovery Systems, Diegem, Belgium) using a perfusion pack with optional oxygenation tubing (reference LKT200X, Organ Recovery Systems, Diegem, Belgium) and KPS-1 solution. Pump pressure was set at 30 mmHg. Active oxygenation with carbogen (95% O_2_/5% CO_2_) for oxygen supplementation. In contrast to clinical practice, the renal vein was also cannulated to enable creating an additional venous loop for continuous venous Po_2_ measurement. Two oxygen microsensors (Flow-Through Cell Housed Oxygen Microsensor, FRCH-PSt11-TF-OIW, PreSens Precision Sensing GmhH, Regensburg, Germany) were added in series to the perfusion circuit at inflow and outflow level of the kidney. According to the study groups two different oxygenation techniques were applied.

#### 2.4.1. Membrane Oxygenation

**Figure 1:**
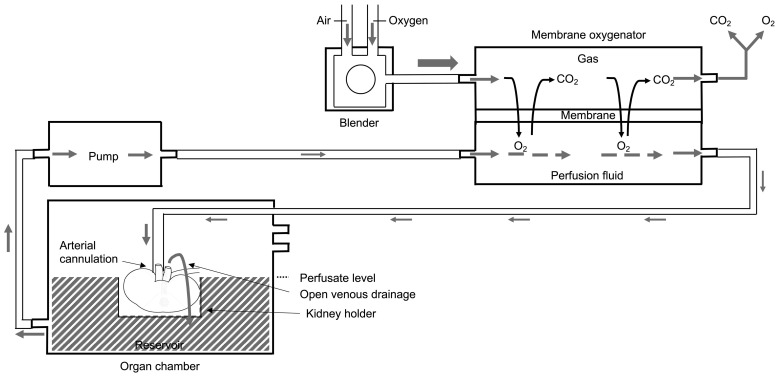
Principles of membrane oxygenation during hypothermic kidney machine perfusion. Between the pump and the organ chamber, a membrane oxygenator is added in series. In the membrane oxygenator, oxygen diffuses from the gas compartment through the thin gas-permeable membrane into the perfusion fluid, and carbon dioxide diffuses from the perfusion fluid into the gas compartment for disposal. By arterial cannulation, the oxygenated perfusion fluid is pumped through the kidney. The reservoir of the organ chamber collects the perfusion fluid via open venous drainage and is pumped again towards the membrane oxygenator. Figure and figure legend slightly adapted but based on Figure 1 of T. Darius et al. [16].

Membrane oxygenation was realized by adding an oxygenator (Dideco Kids D100 neonatal oxygenator, Sorin Group, Mirandola, Italy) and a heat exchanger (used as cooler in this model to prevent warming of the perfusion fluid in the external membrane oxygenator tubes) in series to the perfusion circuit [6,14]. The basic principles of membrane oxygenation during kidney perfusion are illustrated in Figure 1.

#### 2.4.2. Bubble and Surface Oxygenation

**Figure 2:**
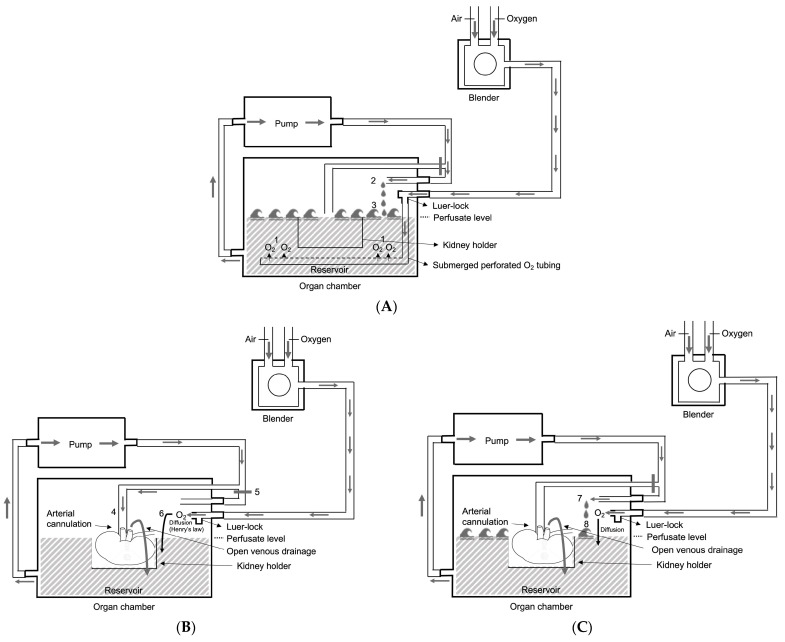
Principles of bubble and surface oxygenation during hypothermic kidney machine perfusion. (**A**) During the entire wash phase, the perfusion fluid is uploaded with oxygen by bubble oxygenation realized by insufflating the carbogen directly to the perfusion solution in the reservoir via a submerged perforated O_2_ tubing segment (1). The perfusion fluid enters the reservoir by a separate wash line (2) and by creating waves the efficiency of this O_2_ uploading process increases (3). (**B**) Surface oxygenation was given at 200 mL/min during kidney perfusion via arterial cannulation with a mean perfusion pressure of 30 mm Hg (4) (and closure of the wash line (5) and removal of the submerged tubing segment of the Luer lock (6)). (**C**) The efficiency of surface oxygenation is enhanced during regularly scheduled wash cycles (7), resulting in breaking the perfusate’s surface layer (8) and increasing the oxygen diffusion in the perfusion fluid. Figure and figure legend slightly adapted but based on Figure 1 of T. Darius et al. [16].

The dissolved O_2_ concentration in the perfusion fluid was raised by bubble and surface oxygenation and the concept is previously described [15,17]. This technique is based on four principles and illustrated in Figure 2. First, bubble oxygenation increases proportionally with oxygen volume and inversely with bubble size resulting in a highly effective O_2_ transfer (achieving perfusate pO_2_ of at least 80–100 kPa (600–750 mmHg) after 15 min of bubble oxygenation) [18]; secondly, the solubility of oxygen is inversely proportional to the temperature [19,20]; thirdly, O_2_ diffuses slowly across the perfusate surface from the gaseous compartment into the perfusion fluid according to Henry’s law (the quantity of O_2_ diffusing into the perfusion fluid will be proportional to the gaseous pO_2_) [21]; fourthly, every 10 min during perfusion, a wash cycle enhances the surface oxygenation efficiency.

### 2.5. Outcome Measures

The primary outcome measures were adenosine triphosphate (ATP) levels at 270 min and at the end of the preservation on tissue biopsies. Secondary outcome measures were pO_2_ during machine perfusion and oxygen consumption, renal flow, and resistance, metabolic perfusate and tissue profile, and classical histological tissue evaluation. 

### 2.6. Samples and Analyses

#### 2.6.1. Oxygen, Renal Resistance, and Flow Measurement during HMP and Perfusate Analyses

Renal resistance and flow are continuously registered by the machine perfusion device. Perfusate samples were taken at start, 30, 60, 120, 180, 240, and 270 min and at the 22 h of HMP. At the inflow and outflow level of the kidney, the perfusate pO_2_ was continuously measured by 2 fiber optic oxygen microsensors and recorded by the OXY-4 micro^®^ (PreSens Precision Sensing GmhH, Regensburg, Germany). A dry chemistry analyzer Fui Dri-Chem NX500 (Fujifilm Corporation, Tokyo, Japan) was used to determine the glucose, aspartate transaminase (AST), and lactate dehydrogenase (LDH) levels. Neat perfusate samples were mixed with a concentrated NMR buffer, granting end concentrations of 0.5 mM DSS, 0.1 M phosphate buffer, 10% D_2_O for metabolite identification and quantification by Nuclear Magnetic Resonance (NMR) spectroscopy. One dimensional proton Proton Nuclear Magnetic Resonance (1D ^1^H-NMR) spectra were acquired on a 600-MHz Bruker Avance III NMR spectrometer in 5 mm NMR tubes. Phase and baseline correction of the spectra were performed with MettaboLabPy software version 0.7.22 (https://ludwigc.github.io/metabolabpy (accessed on 20 October 2022)). Chenomx NMR Suite (Chenomx, Edmonton, Alberta, Canada) was used for metabolite identification and quantification. Metabolic profiles of the perfusion fluid were performed at the start, after 4.5 h, and at the end of HMP. Quantification of the following metabolites was realized: succinate, lactate, glucose, mannitol, acetate, adenine, alanine, aspartate, formate, gluconate, glutamate, glutathione, glycine, and hypoxanthine. Flavin Mononucleotide (FMN) release at each timepoint during HMP was detected by spectrometry (fluorescence filter at 525 nm).

#### 2.6.2. Tissue Sampling and Analysis

At 3 different time-points, wedge biopsies of the kidney were obtained: at start and after 4.5 h of HMP and at the end of the preservation period. Pending metabolic analysis, biopsies were stored fresh at −80 °C, but also fixed in acidified formal alcohol, dehydrated, and embedded in paraffin wax for histology. 

Metabolites (i.e., adenosine monophosphate (AMP), adenosine diphosphate (ADP), adenosine triphosphate (ATP), lactate, succinate, glutamate, FMN and oxidized and reduced nicotinamide adenine dinucleotide (NAD^+^ and NADH, respectively)) were extracted from frozen kidney biopsies by methanol–chloroform extraction and analyzed and quantified by liquid chromatography coupled to electrospray ionization mass spectrometry (LC-ESI-MS) [22,23]. 

In addition, like the perfusate samples, tissue biopsies were also assessed for identification and quantification using Nuclear Magnetic Resonance (NMR) spectroscopy. The following tissue metabolites were quantified: AMP, ADP, ATP, aspartate, gluconate, glutamate, glutathione, lactate, and succinate. 

Histological evaluation by light microscopy was performed after obtaining tissue sections of 4 μm and stained with hematoxylin and eosin. The histopathological consensus criteria for preimplantation kidney biopsies [24] and the acute tubular injury score described by Hosgood et al. [25] were used to assess morphological categories (tubular dilatation, tubular debris, vacuolation, and infiltration).

### 2.7. Statistical Methods

GraphPad Prism 9.5.1 (GraphPad Software, San Diego, CA, USA) was used for data analyzing and charting. Data are presented as mean and standard error of the mean, and comparison of study groups was performed with an ordinary one-way analysis of variance (ANOVA)(or Kruskal–Wallis test in case of non-Gaussian distribution). Comparisons between two unpaired groups were performed by an unpaired *t*-test or Mann–Whitney U test. Statistical significance was set at *p* < 0.05.

## 3. Results

### 3.1. Operative Data and Adverse Events during HMP

Seventeen pigs underwent an uncomplicated bilateral nephrectomy. Nine of the thirty-four HMP-perfused kidneys were excluded from the analysis due to technical-related problems, especially early in the series (unforeseen software problem of the machine perfusion device of the membrane-oxygenated group (*n* = 6), dislocation of the venous cannula (*n* = 1), production error at the tube of the perfusion kit at the level of the air detection resulted in perfusion failure after 4 h 50 of HMP (*n* = 1), and dysfunction of one of the oxygen probes (*n* = 1)). 

### 3.2. Efficacy of Oxygen Administration, Oxygen Consumption and Renal Flow during HMP

**Figure 3:**
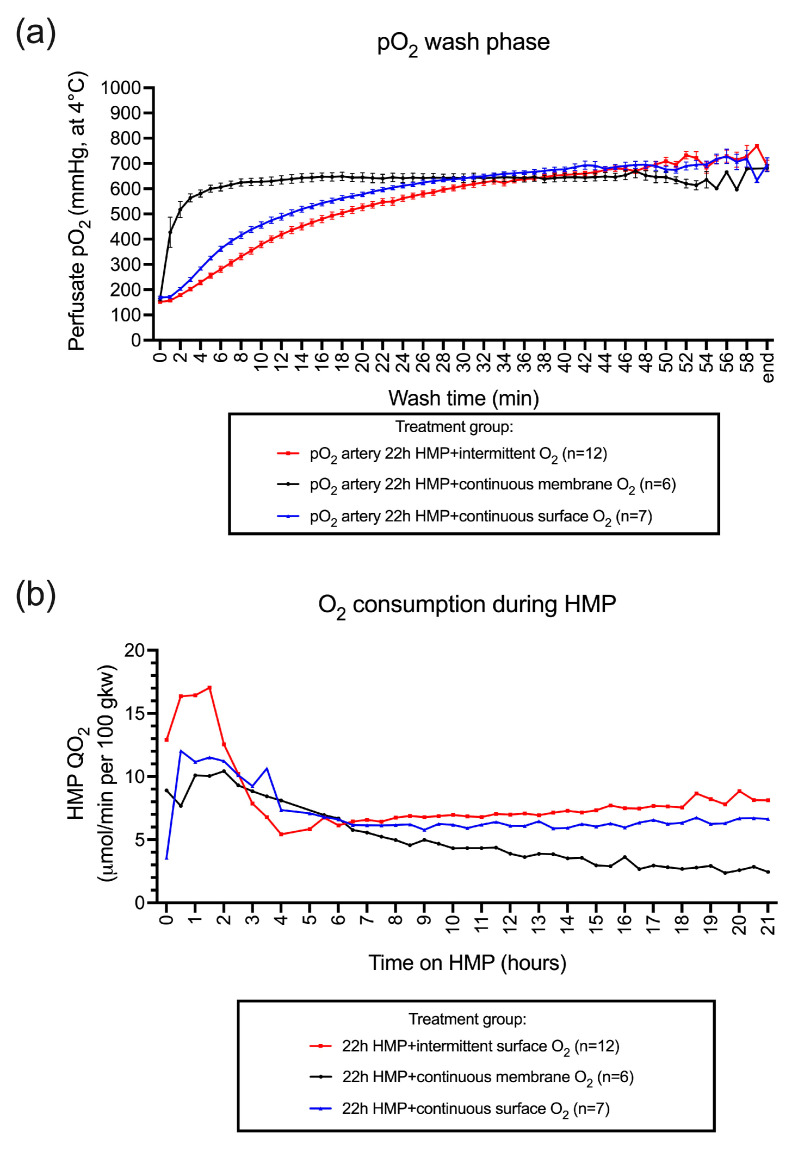

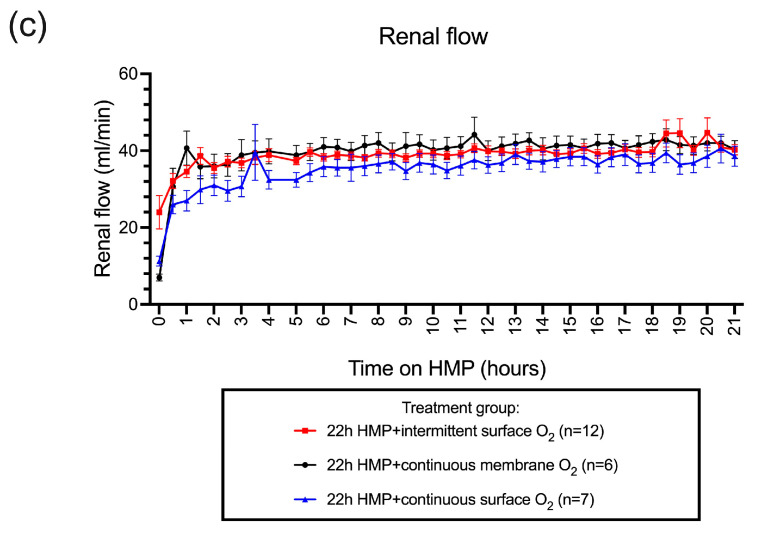
The evolution of perfusate oxygen concentration, O_2_ consumption, and renal flow during hypothermic machine perfusion. To obtain perfusate pO_2_ levels above 500 mmHg (at 4 °C), before connecting the kidney to the perfusion device, short bubble oxygenation of minimum 15 min was demonstrated as efficient as membrane oxygenation (**a**). Both surface-oxygenated groups demonstrated oxygen consumption during the entire preservation period. In contrast, the membrane-oxygenated group demonstrated a decreasing trend of oxygen consumption starting after 2 h of machine perfusion (**b**). Active oxygenation, independent of the type of oxygen administration technique, demonstrated a comparable renal flow profile during the 22 h of machine perfusion (**c**).

To obtain perfusate pO_2_ levels above 500 mmHg (at 4 °C) before connecting the kidney to the perfusion device, direct bubble oxygenation of the perfusion fluid by minimum 15 min was demonstrated as efficient as membrane oxygenation (Figure 3a). Both surface-oxygenated groups demonstrated oxygen consumption during the entire preservation period (Figure 3b). The membrane-oxygenated group demonstrated a decreasing trend of oxygen consumption starting after 2 h of machine perfusion (Figure 3b). Active oxygenation, independent of the type of oxygen administration technique, demonstrated a comparable renal flow profile during the 22 h of machine perfusion (Figure 3c). Accordingly, renal resistance observed the inverse findings. 

### 3.3. Quantification of Metabolite Concentrations in Perfusate

No differences in perfusate glucose, AST, and LDH were detected by a dry chemistry at the end of machine perfusion between all study groups (Figure S1). 

**Figure 4:**
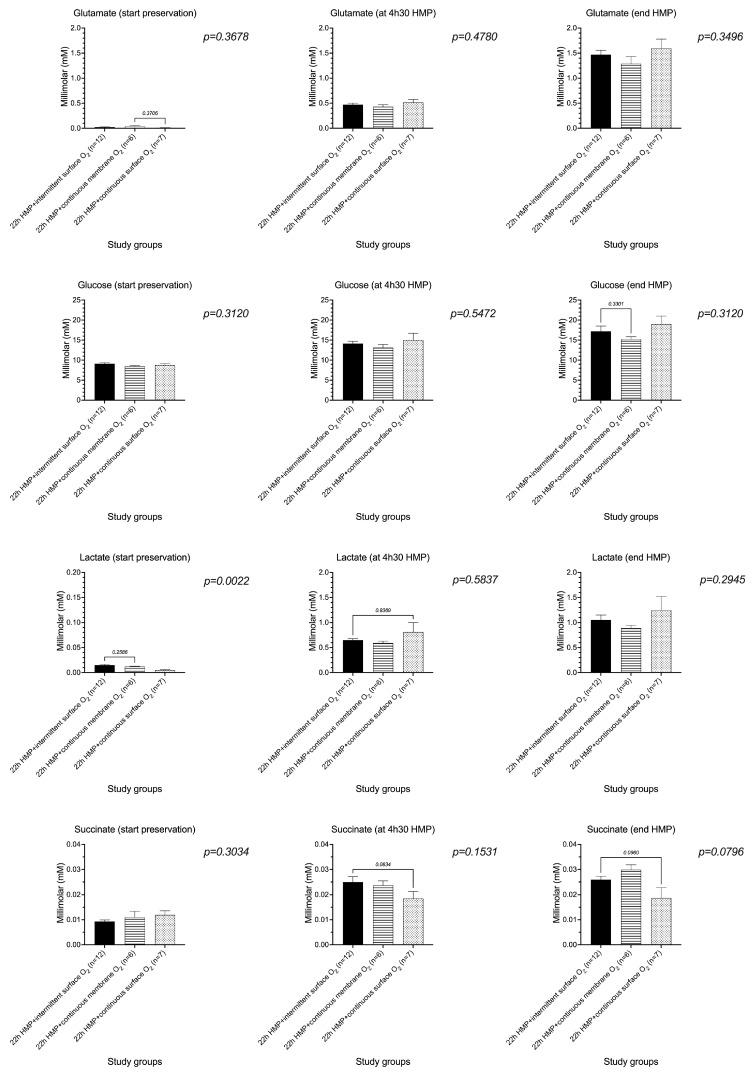
Perfusate concentrations of glutamate, glucose, lactate, and succinate measured by proton nuclear magnetic resonance, demonstrating no difference between all study groups at 4 h 30 and at the end of hypothermic machine perfusion, independent of the duration of oxygenation and oxygen administration technique. HMP, hypothermic machine perfusion; QO_2_, oxygen consumption.

All oxygenation strategies, independent of the duration of oxygenation (intermittent versus continuous) and oxygen administration technique (membrane versus surface oxygenation) during HMP, demonstrated a similar concentration of circulating metabolites glutamate, glucose, lactate, and succinate at 4 h 30 and at the end of the machine perfusion when measured by 1D ^1^H-NMR (Figure 4).

**Figure 5:**
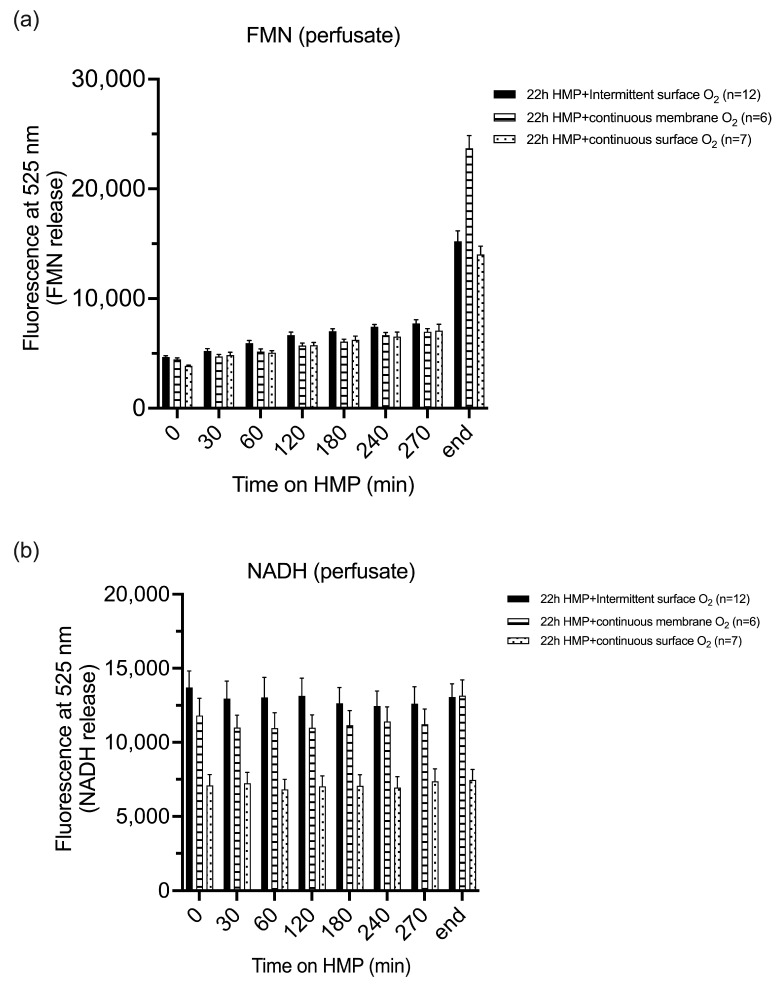
Detection of flavin mononucleotide and NADH release in the perfusate by fluorescence. FMN release was comparable between all study group at 240 and 270 min of HMP. At the end of machine perfusion FMN, values were significantly higher in the continuously membrane-oxygenated group as compared to both surface-oxygenated study groups (*p* = 0.0012) (**a**). At each timepoint, NADH release in the circulating perfusate detected by fluorescence was significantly higher in the intermittently oxygenated HMP group as compared with the continuously surface-oxygenated HMP group but comparable to the membrane-oxygenated group (**b**). FMN, flavin mononucleotide; HMP, hypothermic machine perfusion; NADH, reduced nicotinamide adenine dinucleotide.

FMN release in the circulating perfusate detected by fluorescence was comparable between all study groups at 240 and 270 min of HMP (*p* = 0.0625 and *p* = 0.3663, respectively) (Figure 5a). However, at 60, 120, and 180 min, a significant difference in FMN detection was demonstrated between all study groups (*p* = 0.0260, *p* = 0.0264, *p* = 0.0278, respectively). The intermittently oxygenated study group demonstrated significantly higher FMN values from 60 to 180 min as compared to both continuously oxygenated study groups. At the end of machine perfusion, FNM values were significantly higher in the continuous membrane-oxygenated group as compared to both surface-oxygenated study groups (*p* = 0.0012).

In contrast, NADH release in the circulating perfusate detected by fluorescence, at each timepoint, was significantly higher in the intermittently oxygenated HMP group as compared with the continuously surface-oxygenated HMP group but comparable to the membrane-oxygenated group (Figure 5b).

### 3.4. Quantification of Metabolite Concentrations in Tissue

**Figure 6:**
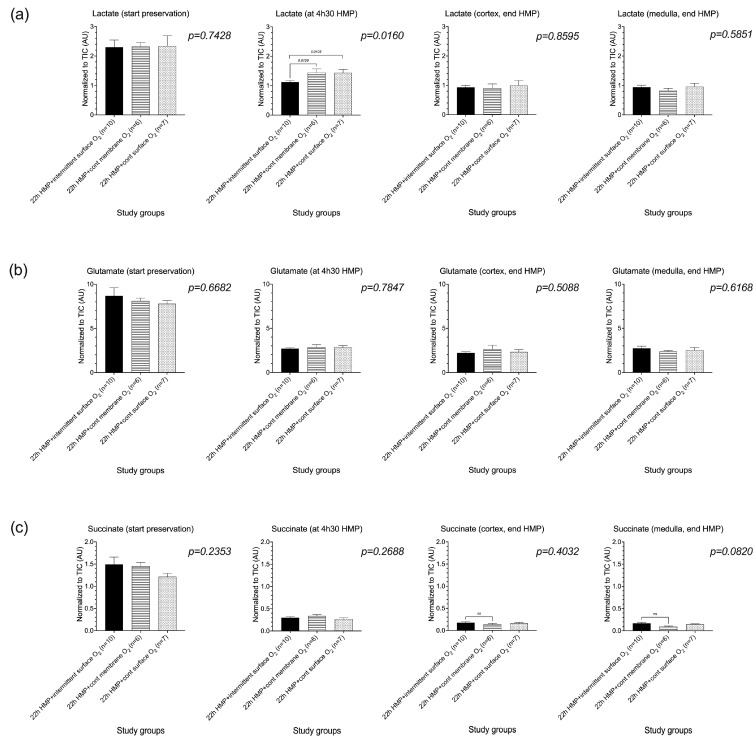

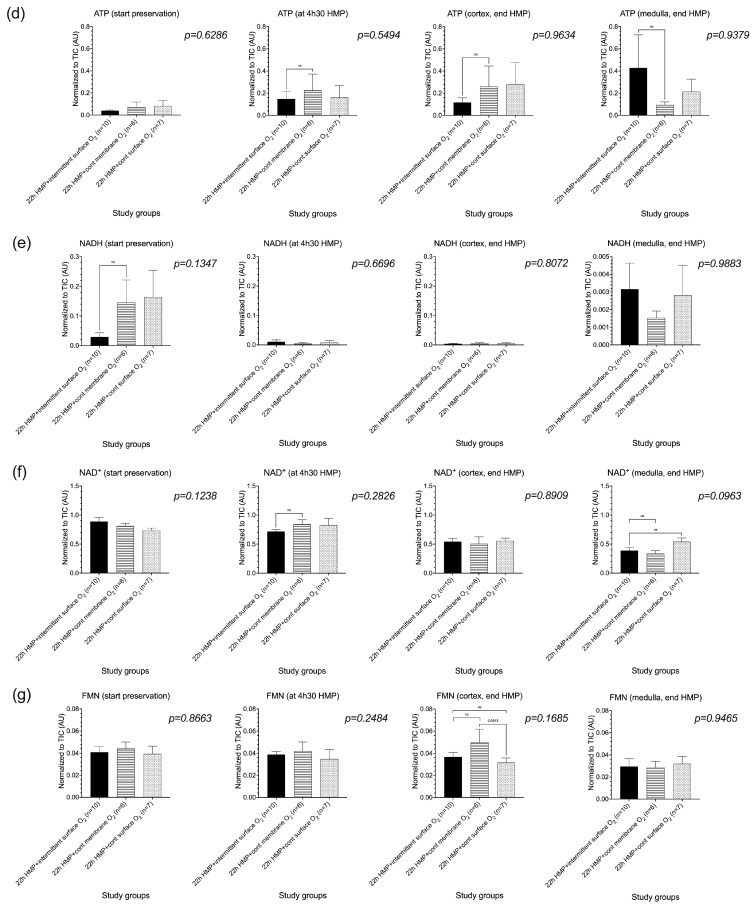
Metabolic tissue analysis by liquid chromatography electrospray ionization mass spectrometry demonstrated that lactate levels measured at 4 h 30 of HMP were significantly lower in the intermittent surface-oxygenated group as compared with both continuously oxygenated groups but were comparable between all groups at the end of the preservation period (**a**). No differences in glutamate, succinate, ATP, NADH, NAD^+^, and FMN level were observed in biopsies obtained at 4 h 30 and at the end of perfusion, comparing all study groups (**b**–**g**). ATP, adenosine triphosphate; FMN, flavin mononucleotide; HMP, hypothermic machine perfusion, NADH, reduced nicotinamide adenine dinucleotide; NAD^+^, oxidized nicotinamide adenine dinucleotide.

Lactate levels measured at 4 h 30 of machine perfusion were significantly (*p* = 0.0160) lower in the intermittent surface-oxygenated group as compared with both continuously oxygenated groups by LC-ESI-MS but were comparable between all groups at the end of the preservation period (Figure 6a). No differences in glutamate, succinate, ATP, ADP, AMP, NADH, NAD+, and FMN level were observed in biopsies obtained at 4 h 30 and at the end of perfusion, comparing all study groups by LC-ESI-MS analysis (Figure 6b–g) (ADP and AMP not shown).

**Figure 7:**
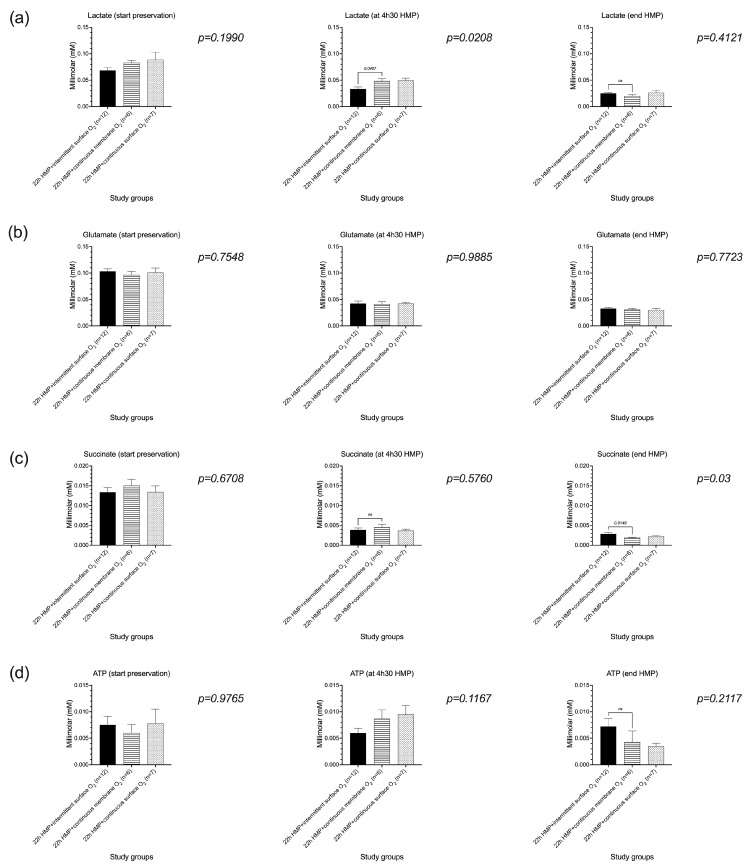

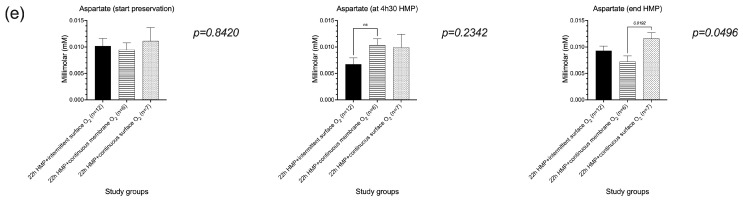
Metabolic tissue analysis by proton NMR demonstrated comparable concentrations of lactate, glutamate, ATP, and aspartate, during and at the end of machine perfusion. At the end of HMP, succinate was significantly higher in the intermittent surface-oxygenated group as compared with the continuous membrane-oxygenated group but comparable with the continuously surface-oxygenated group. (**a**–**e**) Lactate as a marker of anaerobic metabolism as well as the regeneration of ATP, and glutamate and aspartate were comparable between all the study groups at the end of machine perfusion. ATP, adenosine triphosphate; HMP, hypothermic machine perfusion; ns, not significant.

Metabolic tissue analysis by ^1^H-NMR demonstrated similar results on lactate, glutamate, ATP, and aspartate, during and at the end of machine perfusion, as compared with LC-ESI-MS analysis (Figure 7). In contrast with LC-ESI-MS analysis, succinate measured by 1H-NMR was significantly higher at the end of HMP in the intermittent surface- oxygenated group as compared with the continuous membrane-oxygenated group (*p* = 0.0149) but comparable with the continuously surface-oxygenated group (*p* = 0.0650) (see also Figure 7).

### 3.5. Histological Tissue Evaluation

Acute tubular injury at the end of the machine perfusion period assessed by the Hosgood score and Banff criteria demonstrated no significant difference between all study groups (Figure S2). All other Banff criteria demonstrated no differences between all groups.

## 4. Discussion

This study, using an ex situ preservation porcine kidney model, evaluates the impact of a short interruption (4 h, mimicking organ transport) of surface oxygenation during 22 h of HMP as compared to continuous surface and membrane oxygenation. Metabolic tissue analysis (i.e., succinate, ATP, FMN, lactate, glutamate, NADH and NAD^+^), during and at the end of the preservation period, demonstrated a similar mitochondrial protection between all study groups. These observations illustrate that mitochondrial protection during HMP does not necessarily require active oxygenation during the entire machine preservation period. Therefore, intermittent surface oxygenation might be an effective oxygenation strategy to recondition mitochondria during hypothermic machine-perfused kidneys as compared with membrane-oxygenated kidneys and has the potential to reduce the ecological and economic impact of active oxygenation during HMP by eliminating the need for a membrane oxygenator and oxygen source during transport.

Bubble and surface oxygenation is an innovative oxygenation technique as alternative for membrane oxygenation. This oxygenation technique was recently introduced in clinical practice in different European countries applied first with the LifePort Kidney Transporter^®^ (Organ Recovery Systems, Diegem, Belgium) in 2022 [16]. The membrane oxygenator can be omitted during HMP because of the following two reasons. First, current HMP preservation solutions are still acellular and without oxygen carrier. Therefore, oxygen reaches the kidney tissue by diffusion [17]. Secondly, the oxygen transfer capacity of a membrane oxygenator is significantly higher compared to levels needed for sustaining aerobic metabolism of a single kidney at ±4 °C [26,27]. The potential advantages of bubble and surface oxygenation are the following: (1) an effective oxygenation technique, and (2) the economic and ecological costs associated with active oxygenation during HMP could be reduced be eliminating the need for a membrane oxygenator (±400–500 EUR) and oxygen source (±100–150 EUR) during organ transport.

As compared to DBD, kidneys originating from DCD are more susceptible to ischemia-reperfusion injury resulting in a higher risk of DGF, PNF, and graft failure after transplantation [28,29,30,31]. A recent meta-analysis demonstrated the superiority of HMP kidneys compared to SCS, showing reduced rates of DGF and PNF and superior one-year graft survival (OR:1.61 95% CI: 1.02 to 2.53, *p* = 0.04) [32]. In hypothermic conditions (4 °C), oxygen consumption is approximately 5%–10% of that at normal body temperature [33,34,35]. Kidney oxygen consumption during HMP has been demonstrated, with a reported 90% decrease in perfusion fluid oxygen levels after 2 h [8]. This provides the rationale for adding active oxygen during HMP of the kidney to promote oxidative processes, to restore adenosine triphosphate (ATP) debt, and to decrease the ischemic accumulation of mitochondrial succinate a responsible for the harmful effects of IRI [17]. This mechanism is described in detail by Schlegel et al. during different human liver perfusion strategies [36]. Similar pathological processes during kidney perfusion were recently demonstrated by our group [14]. The benefice of active oxygenation in clinical practice in RCTs is currently limited. Active oxygenation during HMP is demonstrated to be beneficial for kidneys originating for DCD donors older than 50 years old when machine perfusion is started immediately after procurement until transplantation, with a decrease in graft failure rate at 1 year as compared with non-oxygenated HMP [10]. In contrast, kidneys originating from DBD, ECD, or DCD donors and reconditioned by oxygenated HMP after preceding static cold storage preservation do not appear to offer any benefit for early graft function [12]. Comparable metabolic tissue analysis (e.g., lactate, glutamate, succinate, ATP, NADH, NAD^+^, and FMN) at 4 h 30 and at the end of HMP in all groups of this study illustrates that the oxygen and ATP debt in DCD kidneys is rapidly corrected after the start of HMP and that interruption of surface oxygenation can safely be halted for organ transport (e.g., Eurotransplant region) to still obtain the same mitochondrial protection at the end the preservation period. This new concept of intermittent oxygenation during HMP is currently being explored in our center by a prospective single center trial (ClinicalTrials.gov identifier: NCT05430620).

Different markers in the perfusion fluid have been assessed and evaluated to predict organ quality and outcome after transplantation [37,38,39,40]. Normothermic machine perfusion was for a long time considered as the only preservation technique qualified for viability and graft function assessment [41,42]. In the Eurotransplant machine perfusion trial, glutathione-S-transferase, N-acetyl-β-D-glycosaminidase, and heart fatty acid-binding protein predicted, independently, DGF, but not PNF and graft survival [43]. According to a systematic review, glutathione-S was the most reliable biomarker for predicting DGF [44]. The limited predictive value and the logistical issues are the main reasons that these markers are not yet applied in daily clinical practice for kidneys. In contrast, the extent of FMN release in the perfusion fluid during oxygenated liver HMP was correlated with functional graft outcome [45]. FMN determination by fluorescence is easy, fast, and cheap, and has the potential to be used as a real-time surrogate marker for mitochondrial injury and to predict ischemia–reperfusion injury. A recent published porcine perfusion model demonstrated the feasibility of FMN measurement in kidneys during oxygenated HMP [46]. FMN quantification was correlated with pre-existing kidney graft injury. In the 60 min warm ischemia (WIT) group, FMN release was significantly higher compared to the 30 min WIT and the control group and also correlated with the damage-associated molecular patters (DAMP) signaling, such as 8-OHdG and HMGB1. ATP replenishment was demonstrated to be the best in control kidneys, followed by 30 min WIT kidneys and finally 60 min WIT kidneys. In this current study, FMN measurement by spectrometry demonstrated no difference at 270 min of HMP between all study groups, illustrating comparable ischemic injury in the intermittent surface oxygenated group as compared with continuous membrane and surface oxygenation. FMN values at 22-h of HMP were significantly higher in the membrane-oxygenated group as compared with both surface-oxygenated groups when measured by fluorescence. In contrast, FMN values on end-preservation tissue biopsies were comparable between all study groups when determined by mass spectrometry. Therefore, the value of perfusate FMN measurement by fluorescence, as a surrogate marker of ischemic injury, needs to be confirmed and correlated with functional outcome in autotransplant models or clinical trials. A previous autotransplant study demonstrated a similar initial graft outcome comparing intermittent surface and continuous membrane oxygenation [15].

The absence of an autotransplant or ex situ normothermic reperfusion model are the main limitations of the current study. However, the correlation of intermittent surface oxygenation with post-transplant outcomes has been confirmed in an autotransplant model [15]. 

## 5. Conclusions

In summary, intermittent surface oxygenation of the preservation fluid during hypothermic kidney machine perfusion is a new oxygenation strategy, and it has the potential to be an effective preservation strategy to support aerobic metabolism, therefore protecting mitochondria and reducing the ecological and economic impact of active oxygenation as compared with membrane-oxygenated kidneys by eliminating the need for a membrane oxygenator and oxygen source during transport.

## Data Availability

Data availability on request.

## Supplementary Materials

The following supporting information can be downloaded at: https://www.mdpi.com/article/10.3390/jcm12113731/s1, Figure S1: Evolution of perfusate glucose, AST, and LDH during hypothermic machine perfusion according to oxygen administration technique and duration of oxygenation (A, B, and C, respectively); Figure S2: Acute tubular injury at the end of 22 h of hypothermic machine perfusion preservation assessed by the Banff criteria (a) and the Hosgood score (b).

## Abbreviations

1D 1H-NMR, one dimensional proton nuclear magnetic resonance; ANOVA, analysis of variance; AST, aspartate transaminase; ADP, adenosine diphosphate; AMP, adenosine monophosphate; ATP, adenosine triphosphate; FMN, flavin mononucleotide; DBD, donation after brain death; DCD, donation after circulatory death; DGF, delayed graft function; ECD, extended criteria donor; eGFR, estimated glomerular filtration rate; HMP, hypothermic machine perfusion; HMPO2, oxygenated hypothermic machine perfusion; LDH, lactate dehydrogenase; NAD^+^, oxidized nicotinamide adenine dinucleotide; NADH, reduced nicotinamide adenine dinucleotide; NMR, nuclear magnetic resonance; LC-ESI-MS, liquid chromatography electrospray ionization mass spectrometry; pO2, partial pressure of oxygen; RCT, randomized clinical trial; SCS, static cold storage; WIT, warm ischemia time.
