# Supplementary material for: Intermittent Surface Oxygenation Results in Similar Mitochondrial Protection and Maintenance of Aerobic Metabolism as Compared to Continuous Oxygenation during Hypothermic Machine Kidney Machine Perfusion
Source: J Clin Med. 2023 May 29;12(11):3731. doi: 10.3390/jcm12113731 (PMC10253557; doi:10.3390/jcm12113731)
Supplement: Supplementary file 1 [file jcm-12-03731-s001.zip › jcm-2343316-supplementary.pdf]

Journal of
Clinical Medicine

MDPI

Figure S1

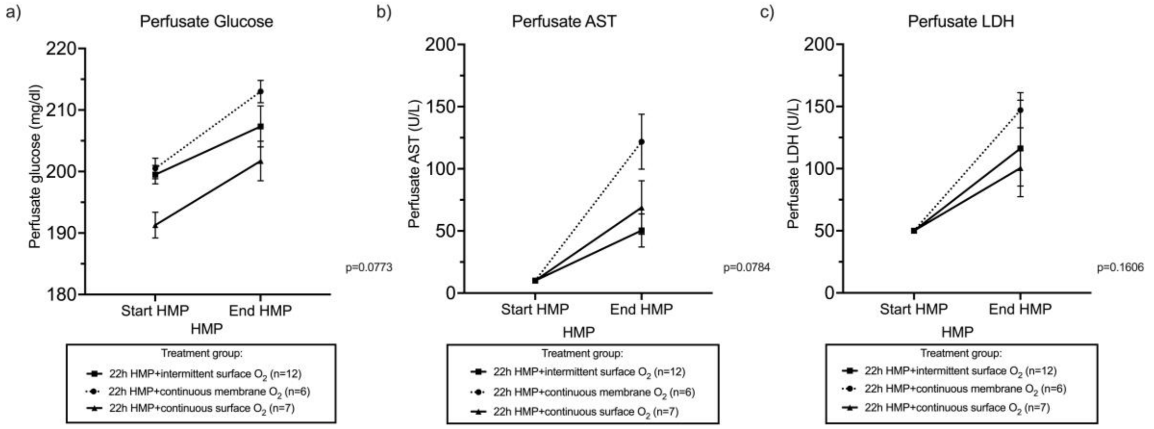

Figure S1. Evolution of perfusate glucose, AST, and LDH during hypothermic machine perfusion according to oxygen administration technique and duration of oxygenation (a, b, and c, respectively). AST, aspartate transaminase; HMP, hypothermic machine perfusion; LDT, lactate dehydrogenase.

Figure S2

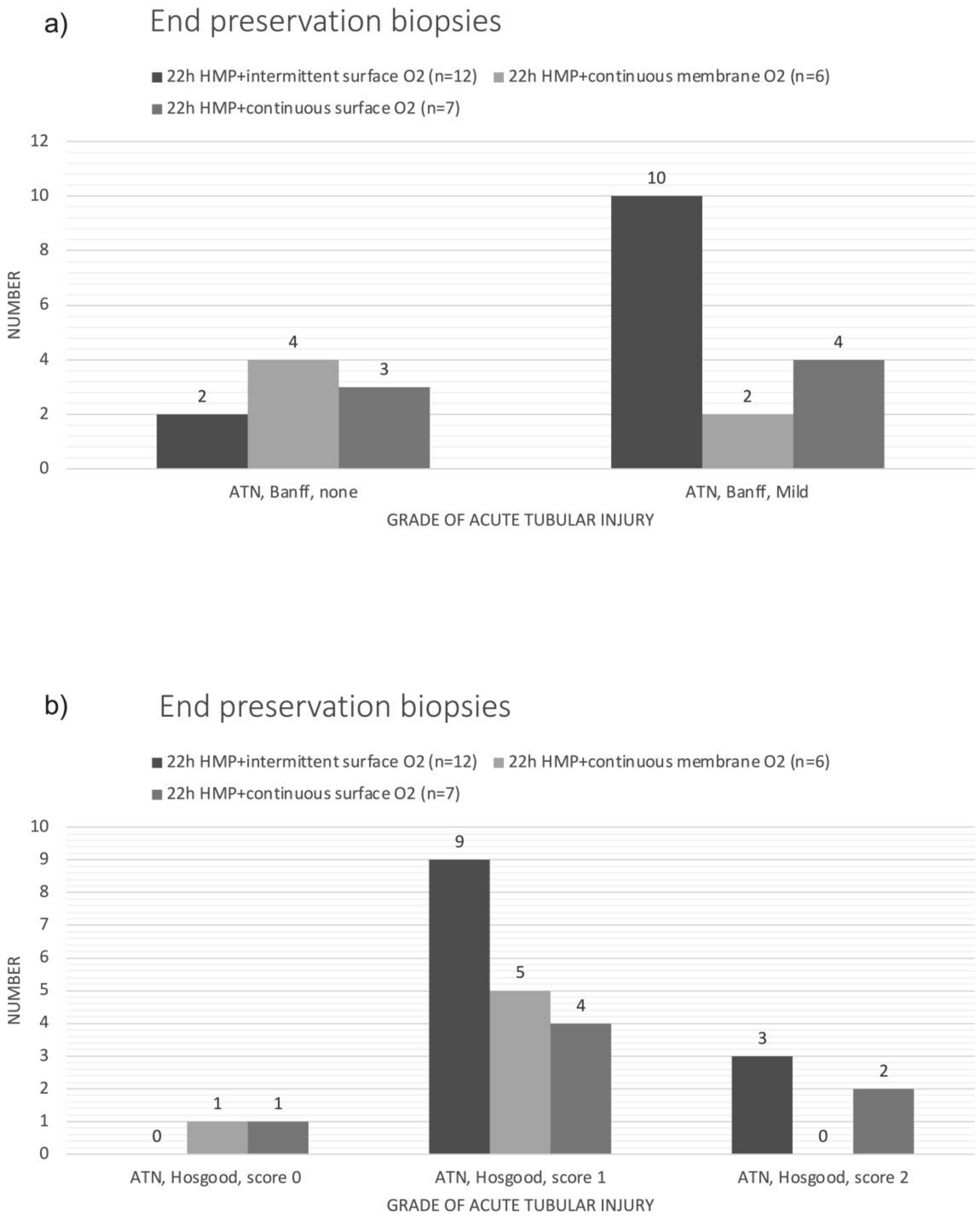

Figure S2. Acute tubular injury at the end of 22 hours of hypothermic machine perfusion preservation assessed by the Banff criteria (a) and the Hosgood score (b) demonstrated no difference between all study groups. ATN, acute tubulus necrosis; HMP, hypothermic machine perfusion; $HMPO_2$, oxygenation hypothermic machine perfusion.
